# Some Considerations of a Wide Subject

**Published:** 1920-01-24

**Authors:** 


### THE PUBLIC WELL-BEING.

### SOME CONSIDERATIONS OF A WIDE SUBJECT.

It is conceivable that a cold tub daily for every individual would do more to settle world discontent than daily meetings of the Peace Conference. This may seem an extravagant way of denoting the profound effect which good health and physical well-being have upon the happiness of mankind, but it defines an essential truth. The political development of the world reacts at almost every point upon national and individual welfare, and it is unnecessary to go beyond the experiences of the past few years to find illustrations of this statement. War affects food supplies, the distribution of coal, and the necessaries of life, the proper exercise of individual liberty, and all these things concern well-being of nations and peoples. It is a social question, an educational question, and an industrial question. So that while the well-being of the world can tone up and vitalise all the arts of good, progressive government, the results of bad, reactionary statesmanship can bring about much individual misery and ill-health.

It is one of the signs of the times that the League of Nations includes in its terrific task many of those subjects which govern the health of nations. In fact, one of its larger aims is to raise the standard of work and living by educating the nations to a better recognition of the world-wide benefits to be derived from it. Conditions of labour in factories, the employment of mothers and children, housing, sanitation, mothercraft, child welfare, the proper organisation and distribution of the necessaries of life, and the whole field of economic politics are involved. A railway strike or a coal strike kills the weak and creates sickness among the strong. A crop failure causes famine and suffering, local government—good or bad—affects every home, and education is at the root of most difficulties. It is almost impossible to extricate any of these things from the body of civilised existence and say that any one of them does not concern public health.

The State has recognised this by taking an extended share in the matter, and we have the controversy of how far State interference is a nuisance. There is the assertion that legislation cannot make drunken men sober, and doubtless controversy will continue as to how far legislation can make the unclean clean or renegades good citizens. The delimitation of State interference is a big question, but it is common ground that the State cannot ignore so vital a part of national life. It has done, and still can do, much to put an end to such matters as the brutality which gives small children long and arduous tasks of labour. It can institute and enforce powers to secure at least a minimum of decency in housing. It can insist on healthy factories. It can endeavour to protect good citizens from the shortcomings of bad citizens. It can safeguard to some extent the purity of foodstuffs. It can educate the public-health sense, and it can play an important part in fighting disease. If good health were a fetish, like sandals in the garden suburbs, no harm would result. But beyond all this, it is the individual who is the final factor. A public authority may issue warnings against the objectionable practice of spitting, but it is worthless if the individual ignores them. There are still people living in hovels though they can afford respectable houses, and other examples might be added.

There is a world of useful discussion and experiment in this great field of national well-being, which is being explored not only by Governments, but also by a large number of voluntary associations which bring zeal and knowledge to the task. The Hospital has in the ordinary course of events given attention to the subject in many aspects, but it hopes in future to devote a special section to it, and invites the co-operation of all those who are in any way interested. The proper understanding and progress of public health ought to run parallel with the progress of preventive medicine in making life happier, healthier, and more full of colour.

